# Obituary

**Published:** 1921-11

**Authors:** 


### OBITUARY

Mathew H. Cryer, D.D.S., M.D. Dr. Cryer died at his home in Landsdown, Pa., August 12, 1921, in the 82nd year of his age after a long illness.

Doctor Cryer is so well known that he needs no encomiums, as everyone knows of his great work to the world as a student, practitioner and author. A splendid tribute to his life and work is printed in the *Dental Cosmos* for October, written by his long and beloved friend Frank L. Hise.

Doctor Cryer's best known work is found in his book "Studies in the Internal Anatomy of the Human Face," published in 1901.

Doctor Cryer was a great teacher, and did a great deal to make a teachable work on oral surgery for dental students. He was an industrious student and worker, and the profession owes much to his faithful and conscientious labors. He gave honor and credit to the dental profession because his devotion was a love of labor for the cause which he loved. How much such men pay for what we get we never realize, but it really costs more than it ought for what we do for them.

